# Fracture Mechanics Method for Word Embedding Generation of Neural Probabilistic Linguistic Model

**DOI:** 10.1155/2016/3506261

**Published:** 2016-09-06

**Authors:** Size Bi, Xiao Liang, Ting-lei Huang

**Affiliations:** Institute of Electronics, Chinese Academy of Sciences, Beijing, China

## Abstract

Word embedding, a lexical vector representation generated via the neural linguistic model (NLM), is empirically demonstrated to be appropriate for improvement of the performance of traditional language model. However, the supreme dimensionality that is inherent in NLM contributes to the problems of hyperparameters and long-time training in modeling. Here, we propose a force-directed method to improve such problems for simplifying the generation of word embedding. In this framework, each word is assumed as a point in the real world; thus it can approximately simulate the physical movement following certain mechanics. To simulate the variation of meaning in phrases, we use the fracture mechanics to do the formation and breakdown of meaning combined by a 2-gram word group. With the experiments on the natural linguistic tasks of part-of-speech tagging, named entity recognition and semantic role labeling, the result demonstrated that the 2-dimensional word embedding can rival the word embeddings generated by classic NLMs, in terms of accuracy, recall, and text visualization.

## 1. Introduction

Word embedding is a word numerical representation that uses a continuous vector space to represent a group of words [1]. In the word vector space, each word corresponds to a unique point. Intuitively, those points that have similar meanings should be put close, while those who are distant in meaning should be put far away. Based on the space, the degree of relation between words can be estimated via computing the distance between vector points, such as the Euclidian or Mahalanobis distance. Such semantic numeralization enables textual and symbol data to be processed in the traditional neural models. For this reason, more nature language processing (NLP) systems are improved with these neural network technologies [2] and enhance the performance of common natural linguistic tasks [3], such as POS (part-of-speech tagging), chunking, NER (named entity recognition) [4], and SRL (semantic role labeling). However, the training for such word embedding is time consuming and required sophisticated tuning for parameters of the model [5], because these models have a huge number of parameters needed for training, which are high dimensional and sparse. Moreover, text visualization for such high-dimensional data suffered the distortion of information from the preprocessing in dimensional reduction, confusing the understanding of data relations [6].

The word vectorization has always been an active topic for text mining and NLP. Their generating approaches can be roughly divided into two categories: manual coding and autocoding. Manual coding is to code words according to the knowledge of domain experts. It is a heavy work for considering the value of each word. For example, WordNet is a huge project whose aim is to build a word graph database by language experts, where word meanings are associated and presented via the tree-based structural graph [7, 8]. Such representation can only associate a few ranges of words for each word. It is insufficient to build a global relation for all words. On the other hand, autocoding is to code word by neural network models [9]. Every word is initialized with a random vector and varied following the parameter tuning according to a range of contexts around a word. Generally, such methods are performed by the training of NLM, where word embedding is a part of result when the convergence of the objective function is finished [1]. However, the NLM-based approaches such as feedforward neural networks [1], recurrent neural network (RNN) [8], and restricted Boltzmann machine (RBM) [10] have also suffered from the high learning complexity [11], sophisticated preferences [12], and the curse of dimensionality [13].

In this article, we present a novel method for word embedding learning that can reduce the high dimensionality, which is inherent in the traditional NLM. We assume that the generation of word embedding can be viewed as a particle movement on a plane. Particles that are close represent the corresponding words have the similar meaning, whereas the particles that are distant represent the corresponding words are far away in meaning. For simulating text semantics as correctly as possible, a fracture mechanics model is presented for controlling the generating process of word embedding. We aim to provide an effective, intuitive approach for learning a 2-dimensional word vector, which is applicable to the general natural language tasks. In particular, we omit the homonymy and polysemy for keeping the consistency of word representation. With this context, each word corresponds to a single vector. The generating model that is based on neural networks is substituted by a particle system, which can simulate the correlating process of semanticity between words.

Our specific contributions are as follows:

- We propose a force-directed model that is based on fracture mechanics to generate word embedding. A linear elastic fracture model is introduced to control the varying progress of word semantic.
- We use a language model that is based on the feedforward NLM to experiment with the word embedding on the task of POS and NER and SRL, where the word embedding is the input of the language model.
- The coordinates of the 2-dimensional word embedding can be used for word visualization that facilitates observing the degree of relation among words intuitively.

The next section describes the related work regarding word numerical representation.  Section 3 introduces our methodology. Sections 4 and 5 give the result and discussion.  Section 6 gives a conclusion and some possible works in future.

## 2. Background

### 2.1. Text Representation by Word Embedding

Choosing an appropriate data representation can facilitate the performing of a machine learning algorithm. The related methods have developed at the level of automatic features selection according to the requirement of applications [14]. As a branch of machine learning, representation learning [15] has gradually become active in some famous communities, especially to investigate knowledge extraction from some raw datasets. Text representation in natural linguistic tasks can be divided into the three levels of corpus [16, 17], paragraph [18, 19], and word [20–22]. This paper focuses on the representation learning for words. Feature words and context have been considered as the foundation for text representation learning and the constructing of a NLP system [23–25]. We follow this direction aiming at mapping word text to a vector space. Compared with the representing level of paragraph and corpus, word is the fewer granularities of semantics that is more suitable to be analyzed by a vector distance.

The idea that uses a vector space to map word meaning is proposed from [26] initially. The earlier representation learnings fail to consider the semantic measurement for the differences between words and emphasize how to quantize feature words. For example, in [27], to represent the word “dove,” the first byte of the corresponding representation denotes the property of its shape that is set to “1,” if the dove satisfies some conditions. The representation learning did not focus on the context feature but presents some approaches for measuring the differences regarding word semantic. The self-organizing map (SOM) [27] is employed to compute word vector distance, which uses the length to represent the neighboring degree of word meanings. Based on SOM [28], high frequency cowords are mapped to a 90-dimensional vector space. The investigation [29, 30] for SOM-based word vector applies it in the fields of NLP and data mining.

### 2.2. Generation of Word Embedding

Word representation has started to integrate some measuring approaches for text semantic with the developing of neural networks in probabilistic linguistic model. Reference [31] proposed to use neural network to build a language model. There exits an assumption that the numerical result of those similar meaning words should be put closely, whereas the result regarding those distant meaning words should be put far away. Meanwhile, the probability function is introduced to add the probability output for NLM, which can give a statistics result for estimating a given *n*-gram words combination. In the proposed models, a convoluted neural network [3] that is tuned by hand somewhat after learning obtains an accuracy of 97.20% in POS, 93.65% in chunking, 88.67% in NER, and 74.15% in SRL. A comparison between conditional restricted Boltzmann machine models and support vector machines [32] is performed for a music annotator system, which is based on the context around lyric that can use cooccurrence and sequential features to improve the accuracy of labeling. The SENNA software [3, 33] performs a word sense disambiguation with an accuracy of 72.35%. Word representation is denoted by multiple vectors [4] to express the polysemy that is tested on NER, which shows that it rivals the traditional linguistic models. In addition, word representation learning has been trained based on the *n*-gram models, hidden Markov models, and partial lattice Markov random field model [34].

For representation learning by NLM, [1] proposes a feedforward neural network to train the word embedding, which is regarded as the internal parameters requiring the tuning following the object function. Reference [35] presents a text sequence learning model that is called RNN, which is capable of capturing local context feature in sequence of interest. Following this route, more machine learning algorithms are introduced to improve the weaknesses of natural linguistic tasks. Reference [10] uses the restricted Boltzmann machine to improve the performance of the sequential probability function. Reference [36] creates a hierarchical learning that represents the semantic relation among words as a tree structure. The deep learning mechanism is tried to build a NLM [37].

### 2.3. Force Simulated Method

The force-directed algorithms are mainly applied in data visualization. Reference [38] compares several force-directed algorithms. Reference [39] uses these methods for analyzing a complex social network, which adds a gravity to draw the graph of social network. In some cross-domain applications, wireless sensors network uses it to build layouts [40], and [41] performs electrical circuit layouts automatically based on the force rules.

The reason why we use the force simulated approach to improve the generation of word embedding is that the relation between words semantic is relatively stable within a certain numbers of documents that depict the similar topic. This is somewhat like the convergence of the stable energy status in the force-directed algorithm. We are inspired it to use the idea of the force-related methods to improve the problems of NLM.

## 3. Methodology

### 3.1. Word-Particle Model

To map word into a particles system, we must define a mapping rule that specifies which attribute of particle corresponds to which feature of word semantics. The linking table is shown in Table 1. The table consists of two parts, where the left part designates the names of each property for particles and the right part gives the explanation for the corresponding semantic feature.

For more explanation, we explain them one by one.

(i) ID, each word has a unique identifier for relating the corresponding particle.
(ii) Pos, it is exactly the attribute that we want to train, which is also called word embedding that denotes the coordinate of a particle in a plane actually.
(iii) Mass, we use the concept of particle mass to denote the occurrence frequency of a word.
(iv) TmpMass, we use the temporal mass of particle to represent the coword frequency.
(v) Current_Mass & history_Mass & base_Mass, they represent the current mass, historical mass, and basic mass of a particle, respectively. A function combined with them that is used to control the occurrence frequency of coword within a sampling period is described as
  (1) $$\begin{array}{c} Mass=base\_Mass+\frac{history\_Mass}{2}+\frac{Current\_Mass}{2}. \end{array}$$
 For controlling the intensity of relevance between words, we use the concept of edge in physic to describe it.
(vi) Chain, it is an array for recording the ID of the backward relating word.
(vii) Max_flaw, it is the maximum associating strength regarding a group of coword.
(viii) Flaw, it describes the associating strength regarding a group of cowords.
(ix) Radius, it is the repulsion radius that keeps a minimum distance from other word-particles.
(x) Pull_Radius, it is the pulling radius, which means if other word-particles break in, they will be pushed away keeping the radius distance from the intruded word-particle.
(xi) Velocity, it defines a semantic shifting trend that can strengthen the relation of two force-affected words.

**Figure 1:**
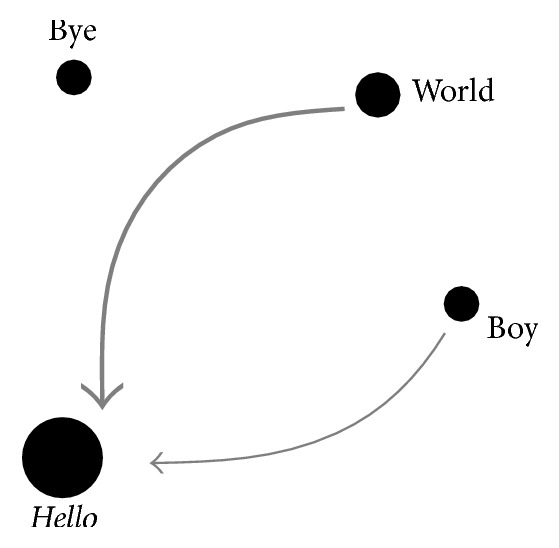
An example of the word-particle model.

In Figure 1, the word-particles world and boy are backward related to hello, and the word-particle bye is an isolated word-particle. The intensity of relation between the word-particles hello and world is stronger than the intensity of relation between hello and boy. The directed line denotes the direction of word order, where the pointed word-particles can drive their linking word-particles. For example, the coword hello world appears more frequent than the coword hello boy.

### 3.2. Semantic Relation Building

Based on the word-particle model, we define the semantic relating rule to control the motion of particles within a given text context. The documents for training play the role of a driven-force source, making the words have more opportunities to come together, which appear in similar contexts. The procedure is as follows.

**Step 1 .**

**Figure 2:**
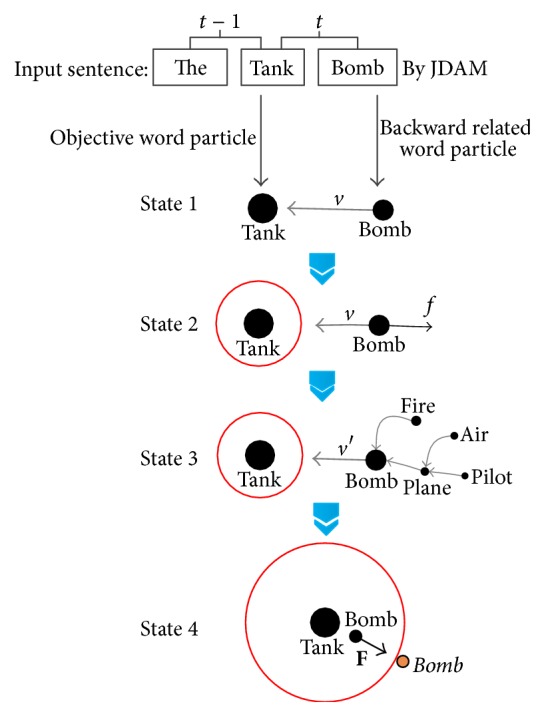
The semantic relating rule.

The word embedding is trained by the document. Each document will be sampled sentence by sentence via a 2-gram window. In the 2-gram window, the first word is assumed as the target object, and the second word is assumed as the associated object. The assumption means that the associated word-particle will be forced to move towards the target word-particle. The related word-particle will be given an impulse. This can drive the word-particle with a certain velocity. The progress is illustrated as state 1 in Figure 2. The word-particle bomb is associated with tank moving with the velocity *v*.

**Step 2 .**

Given an impulse, the word-particle can be initialized with a velocity. Meanwhile, it will be slowed down by the force that comes from friction, until its velocity reduces to zero and does not get in the repulsion radius of its objective word-particle. When the word-particle moves into the repulsion radius of the objective word-particle, it will be stopped at the edge keeping a distance of repulsion radius from the objective word-particle. This is shown as state 2. A velocity affects the word-particle tank, and the word-particle bomb is affected continuously by the friction force *f*.

**Step 3 .**

During a certain period of document learning, some word-particles will set up some relations with other word-particles. We establish a chain reacting rule for simulating the context feature. The rule specifies the impulsion transition in the way of particle by particle, and the initial energy will degrade at each reaction. This passive action simulates the phenomenon that a topic word in a document has more semantics and can be an index for document retrieval. The progress is controlled by (2). Given *m*_0_ denotes the property Mass of the impacted word-particle and *m*_*i*_ denotes this property of other word-particles. The relation-building condition is

(2) i∈Chain,di>Pull_Radius,

where *i* denotes the ID of the *i*th word-particle that relates to the object word-particle, and *d*_*i*_ denotes the corresponding distance of the *i*th word-particle between the object word-particles. The velocity *v*_*t*−1_ will update *v*_*t*_ via (3), if the word-particle satisfies the condition. This procedure will repeat iteratively till the velocity falls to zero. For example, in state 3, the word-particle bomb has two backward associating particles fire and plane. Its initial velocity will be decomposed with plane according to (3), if given an impulsion towards tank. But the word-particle fire fails to move, because it is outside of the Pull_Radius distance of bomb. The decomposition will be delivered repetitively if the velocity fits the condition and is greater than zero.

(3) $$\begin{array}{c} m_{0}v_{t-1}=\left(\;\overset{k}{\underset{i\in Chain,\;\;d_{i}>Pull\_Radius}{\sum }}m_{i}+m_{0}\;\right)v_{t}. \end{array}$$

**Step 4 .**

We add a repulsion radius for keeping the uniqueness of every word-particle, because each word embedding is unique. When the moving word-particle intrudes the repulsion radius of other particles, it will stop and stay at the edge of the affected word-particles, keeping a repulsion radius distance. The progress is shown as state 4. Generally, the word relation network is expected growing stably; we present an inspecting criterion to check the convergence, which is as follows:

(4) $$\begin{array}{c} v_{t}=\frac{m_{0}v_{t-1}}{{lim}_{k\to \infty }\sum_{i\in Chain,\;\;d_{i}>Pull\_Radius}^{k}m_{i}+m_{0}}⟶0. \end{array}$$

In (4), the initial velocity will trend to zero with the relation increasing of the number of associated word-particles; that is, *v*_*t*_ → 0. When the movement of an activated particle becomes tiny, such convergence means the property Pos has reached relatively fixed coordinates. This indicates that the word already has situated in a relatively stable semantic network.

### 3.3. Semantic Relation Separating

**Figure 3:**
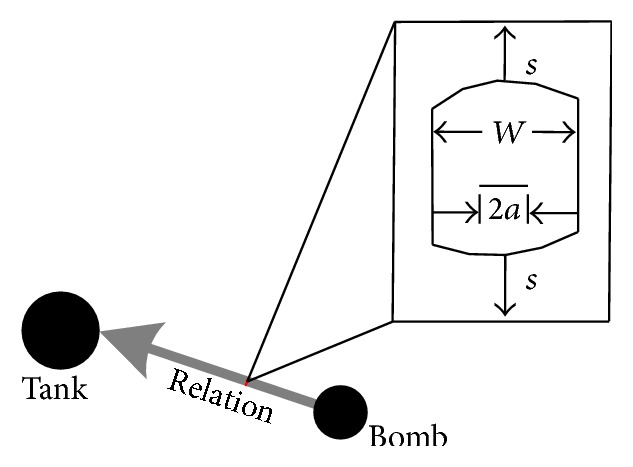
The flaw model.

For controlling the growth of words association, those words that are of low frequency in 2-gram context should be filtered, whereas those words with high frequency of relations should be retained. We propose to use a linear elastic fracture mechanics to control such filtering. A rope model is presented to represent the coword relation, which can be assumed as a flaw that comes from a type of material. The strengthening or weakening of a relation between words is controlled via the corresponding flaw size. An illustration is shown in Figure 3.

More formally, given *W* denotes the width of rope, its value is obtained through the counting of a 2-gram sampling. Its maximum value corresponds to the property Max_flaw. Given 2*a* denotes the size of a flaw corresponding to the property Flaw. Given *s* is the pull force that is calculated by *s* = Mass × Velocity. We use the stress-intensity factor *K* to control the size of a flaw. *K* can be obtained as follows:

(5) $$\begin{array}{c} K=m_{relation}v\sqrt{\pi a}\frac{2a}{W}. \end{array}$$

In (5), the variant *m*_relation_ corresponds to the property regarding the synthetically occurring frequency of a word-particle, and *v* corresponds to the velocity of an activated word-particle. The value of *K* is in proportion to the size of 2*a*, which refers to the concept of flaw. Moreover, the flaw extending speed is denoted by *da*/*dN* as

(6) $$\begin{array}{c} \mathrm{lg}\frac{da}{dN}=\mathrm{lg}C+n\mathrm{lg}\Delta K. \end{array}$$

In (6), lg⁡*C* denotes a compensation constant, and *n* is a scale factor. *da*/*dN* is in proportion of *K*. The condition is that *K* will decrease if *W* goes beyond 2*a*. When the size of flaw is up to *W*, a separation will happen in the semantic relation. This means the associated word-particles are no longer affected by the initial impulses, which are generated from their objective word-particles.

## 4. Simulations

In this section, we compare the proposed word embedding with the three classic NLM-based word embeddings, Huang 2012 [42], C&W [3], and M&S-Turian [20]. Huang 2012 is the word embedding that uses multiple embeddings per word. C&W is the embedding that is trained by a feedforward neural network, and M&S-Turian is the embedding that is obtained by an improved RBM training model. The two datasets Reuters 21578 and RCV1 are used for training and evaluation. In Reuters 21578, we extracted 21,578 documents from the raw XML format and discarded the original class labels and titles, only using the description section. The RCV1 contains 5,000 documents that are written from 50 authors. 70 percent of random sampling among these documents was used to train the word embedding, and the remainder was used to evaluate the NLP tasks of POS, NER, and SRL with other three types of embedding. All words will keep their original forms, whereas the numbers, symbols, and stop words are kept to be trained together. Those words that are not included in training corpus will be discarded. We regard these tasks as a classification problem and apply a unified feedforward neural linguistic model to perform the tasks. The compared word embeddings are readymade, but the parameters of the neural networks require to be trained by the corresponding embedding. We use the benchmark that is provided by [3]. The results regarding the NLP tasks are compared based on this benchmark.

### 4.1. Evaluation

The benchmark is measured in terms of precision, recall, and *F*1 [3, 20, 42]. Assuming *N* words are waiting for labeling, there exit {*N*1∣*N*1 ≤ *N*} words that are labeled correctly and {*N*2∣*N*2 ≤ *N*} words that are labeled wrong. The value of precision is used to evaluate the accuracy of labeling on POS, NER, and SRL. The precision can be obtained as

(7) $$\begin{array}{c} p=\frac{N_{1}}{N_{1}+N_{2}}. \end{array}$$

The value of recall is used to evaluate the coverage of labeling on POS, NER, and SRL. The recall can be calculated as

(8) $$\begin{array}{c} r=\frac{N_{1}}{N}. \end{array}$$

The *F*1 is a combining evaluation with precision and recall, which are as follows:

(9) $$\begin{array}{c} F1=\frac{2pr}{p+r}. \end{array}$$

### 4.2. Training

**Figure 4:**
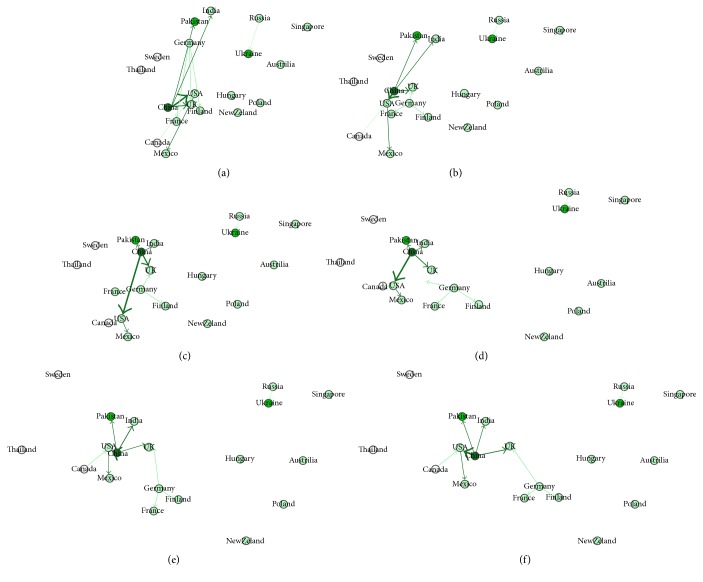
Force-directed embedding for country names.

The parameters of the physical system are set as follows. The coefficient of fiction is set to 0.1, the coefficient of gravity is set to 9.8, and the initial velocity is set to 2. The parameters that control semantic separating are set such that Max_flaw is set to 0.2, the initial value of Flaw is 0, Radius is set to 1, and Pull_Raduis is set to 20. For controlling the flaw extending speed, lg⁡*C* is set to 1 and *n* is set to 1. We demonstrate the training result in terms of the generating procedure of word graph and average speed of word-particles. A word graph can give an intuitive visualization for observing a group of word relations. We test a small number of datasets to simulate the word embedding generation. The result is shown in Figure 4, which contains the names from 19 countries.

In Figure 4(a), all the words appear in the plain physical system and obtain a certain position, because the training documents had given some forces to direct the word arranging that follows the context in original text. But in this stage, some word-particles still have a certain degree of speed to move. Those frequent word-particles such as China, USA, Germany, and France have a relatively high speed; they move pulling their backward relating word-particles (Figures 4(b)–4(d)). For example, China has four backward relating word-particles, Pakistan, India, USA, and UK; Germany has two backward relating word-particles, France and Finland. The other isolated word-particles have situated in a relatively stable position with a few movements. We can see that some word-particles overlay with each other (Figure 4(d)); for example, India and Pakistan are too close at China, and Canada overlays USA. The repulsing force starts to function at this time. The too close word-particles will push each other until they reach a balance distance (Figure 4(e)). When the inputting documents are all about similar topics, the positions of word-particles will also not vary too much, showing a relatively stable topological graph (Figure 4(f)).

**Figure 5:**
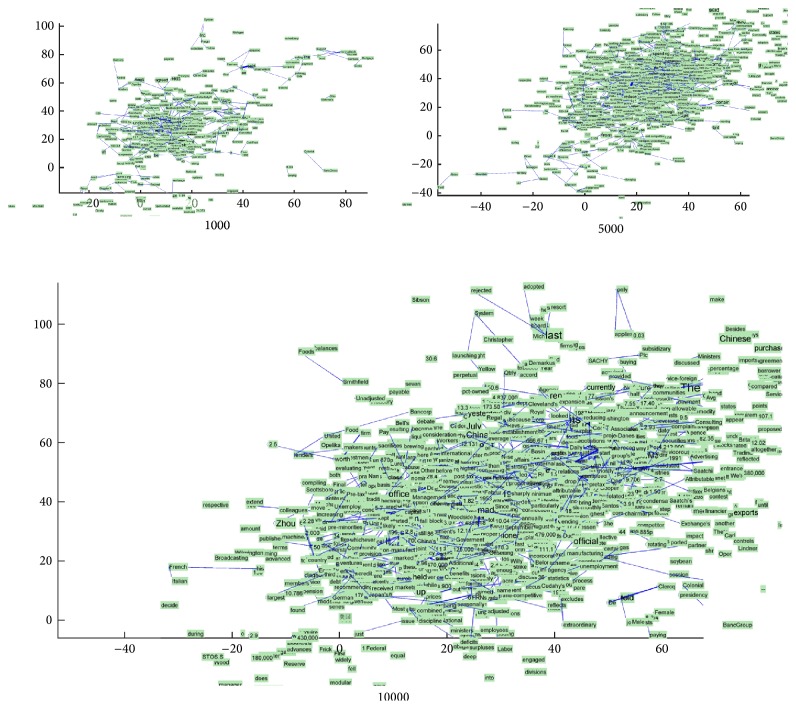
Force-directed embedding for Reuters 21578.

The training result of dataset Reuters 21578 is shown in Figure 5. Each word-particle is colored with a green block. The intensity of the relation between word-particles is represented by a blue line, where the thicker lines mean a higher frequency of coword relation. The position of each word-particle is a 2-dimensional coordinate that is exactly the training result word embedding. The result shows that the numbers of word relations and new word-particles will grow with the training, which iterates from 1,000 to 10,000. The particles system expands the region outward gradually. The particle distribution presents as an ellipse for accommodating more new words-particles.

**Figure 6:**
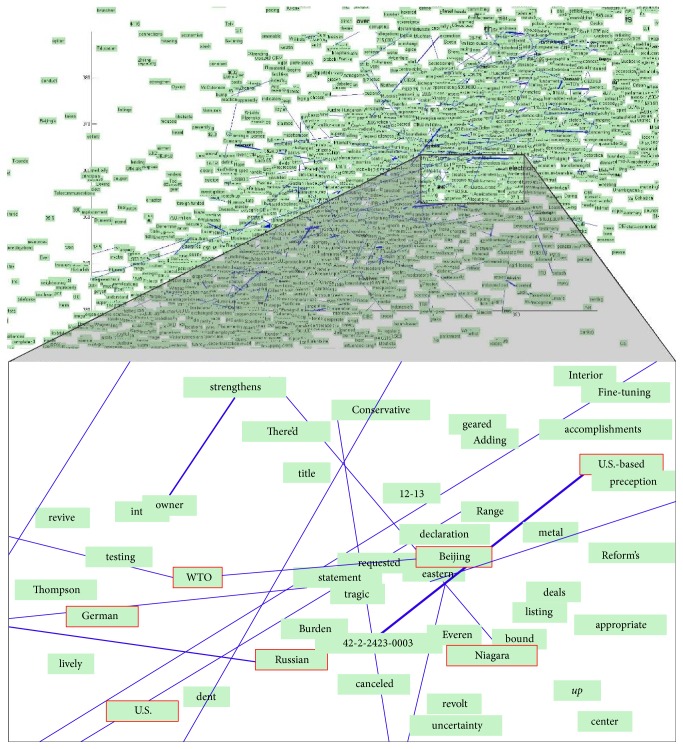
Force-directed embedding for RCV1.

The training result of RCV1 is shown in Figure 6. Such distance-based semantic measurement can be interpreted from some viewpoints. For example, the country and geographical word-particles, German, Russian, U.S., and Beijing, are clustered together. The geo-related word-particles, Niagara, WTO, and U.S.-based, are pushed closely to these words. Such word-particle graph can present an intuitive result for evaluating the training of word embedding; no matter during the training or after training, we can intervene the position of word-particle to improve the final result in a data-visualization based way.

**Figure 7:**
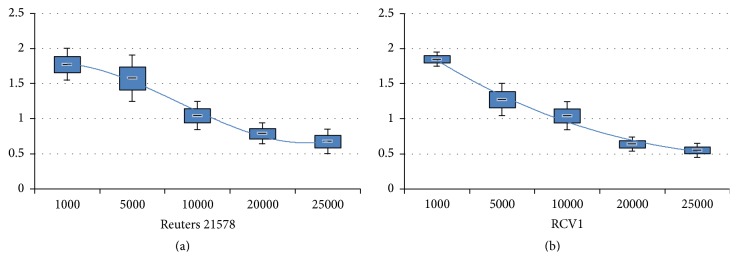
The *y*-axis represents the velocity of a word-particle; the *x*-axis represents the number of documents. Both of them show the downwards trends.

On the other hand, we use (3) to estimate the average velocity of word-particles for evaluating the training process from a statistics viewpoint. When the velocity decreases to 1 below, the convergence is assumed to be happening and the assumption coincides with the reality roughly. We experiment 50 times for the two datasets, respectively. The result is shown as the boxplots in Figure 7. From the downwards trends of average velocity, the assumption that a word-semantic network will be stabilized in a certain number of similar documents coincides with the result of two datasets roughly. Both the convergences of the two datasets' training appear at the training stage around the 20,000th documents.

In the presented word embedding learning, there is not a specific converging criterion for terminating the training, because the object functions of these neural based models are nonconvex so there may not exist an optimum value theoretically. Empirically, these word embedding learning methods require repeating documents 20 or 30 times [3]. It brings a serious problem that time consumption is proportional to the number of documents. Such procedure usually requires undergoing a long-term training time [3, 30]. But in our proposed model, we demonstrate that setting a semantic convergence condition is more convenient to select than those neural based approaches. The convergence criterion provides a more explicit direction for word embedding learning. Meanwhile, the result demonstrates that to learn an acceptable word embedding requiring a certain number of documents, small or medium scale datasets may not be appropriate.

## 5. Reuters 21578 and RCV1

For testing the usability, we compare the word embedding with other three word embeddings on the NLP tasks, using the same type of neural linguistic model. The testing items are performed on POS, NER, and SRL. The results are listed in Tables 2 and 3. In Reuters 21578, the labeling system using our word embedding obtains 91.0% on POS, 83.4% on NER, and 67.3% on SRL for *F*1. This *F*1 score gets the third place in POS and the second place on both NER and SRL. In RCV1, it achieves 89.1% on POS, 82.1% on NER, and 65.9% on SRL for *F*1. The *F*1 scores obtain the second place in POS, the third place in NER, and the second place in SRL.

The performance of the proposed word embedding is close to the best results in [3], but the dimensional number is two, which is far less than the 50- or 100-dimensional word embeddings [3, 43]. This brings a benefit for reducing the number of neural cells in performing NLP tasks by such type of linguistic models. Implementing these NLP tasks, we construct a 3-layer feedforward neural network with a 5-cell inputting layer and a 100-cell middle layer and a 25-cell outputting layer. To utilize the compared word embeddings, the number of the inputting vectors is set to 500, because all of them are the 100-dimensional embeddings. But our corresponding inputting layer just requires 10-dimensional vector. The structure of model is simplified which can reduce the complexity of neural networks. This advantage will improve the performance of such models, such as reducing training time and improving the speed of labeling. The result also demonstrates that learning a group of word embeddings cannot be high dimensional and depend on the neural network based approaches. It means word representation learning and the task system constructing can be decomposed to two individual parts. The two-step framework could achieve the same goal with the all-in-one models [3].

## 6. Conclusions and Future Work

In this paper, we propose a force-directed method that uses a fracture mechanic model to learn word embedding. The result demonstrates that the physical simulation approach is feasible. It improves the procedure of the traditional NLM-based approaches in terms of parameters training and tasks performing (POS and NER and SRL). The next works are as follows. The model will be improved to suit streaming data: using a one-step solution for predicting the coordinate of word-particle, which will improve the performance of our system; packaging the properties of word-particle with the  .gefx file format (Graph Exchange XML Format) that can provide a capability for data sharing across multiple data visualizing tools, for example, Gephi.

## Figures and Tables

**Table 1:** Properties of word-particle.

Name	Notes
ID	Identifier
Pos	Coordinates (word embedding)
Word	Corresponding word
Mass	Synthetic aliveness
TmpMass	All backward related particle aliveness
history_Mass	Historical aliveness
Current_Mass	Current aliveness
Base_Mass	Identifier
Chain	Backward related index
Max_flaw	Backward related degree
Flaw	Current flaw length
Radius	Repulsion radius
Pull_Radius	Tension-start radius
Velocity	Current velocity of particle

**Table 2:** Comparison of POS/NER/SRL on Reuters 21578.

	Precision	Recall	*F*1
*Force-directed*	*92.5/84.1/70.2*	*89.5/82.7/64.6*	*91.0/83.4/67.3*
Huang 2012	94.2/86.2/74.8	93.8/86.8/74.1	94.0/86.5/74.4
C&W	93.6/82.4/67.8	92.8/81.5/65.8	93.2/81.9/66.8
M&S-Turian	91.4/82.1/63.6	86.2/75.2/62.5	88.7/78.5/63.0

**Table 3:** Comparison of POS/NER/SRL on RCV1.

	Precision	Recall	*F*1
*Force-directed*	*90.1/82.7/66.3*	*88.2/81.5/65.5*	*89.1/82.1/65.9*
Huang 2012	88.4/83.2/71.3	90.7/84.6/70.3	89.5/83.9/70.8
C&W	88.5/83.5/64.6	85.2/82.6/63.1	86.8/83.0/63.8
M&S-Turian	90.8/80.5/63.6	90.3/73.9/65.7	90.5/77.1/64.6
